# Can Life Begin on Enceladus? A Perspective from Hydrothermal Chemistry

**DOI:** 10.1089/ast.2016.1610

**Published:** 2017-09-01

**Authors:** David Deamer, Bruce Damer

**Affiliations:** Department of Biomolecular Engineering, Baskin School of Engineering, University of California, Santa Cruz, California.

## Abstract

Enceladus is a target of future missions designed to search for existing life or its precursors. Recent flybys of Enceladus by the Cassini probe have confirmed the existence of a long-lived global ocean laced with organic compounds and biologically available nitrogen. This immediately suggests the possibility that life could have begun and may still exist on Enceladus. Here we will compare the properties of two proposed sites for the origin of life on Earth—hydrothermal vents on the ocean floor and hydrothermal volcanic fields at the surface—and ask whether similar conditions could have fostered the origin of life on Enceladus. The answer depends on which of the two sites would be more conducive for the chemical evolution leading to life's origin. A hydrothermal vent origin would allow life to begin in the Enceladus ocean, but if the origin of life requires freshwater hydrothermal pools undergoing wet-dry cycles, the Enceladus ocean could be habitable but lifeless. These arguments also apply directly to Europa and indirectly to early Mars. Key Words: Enceladus—Hydrothermal vents—Hydrothermal fields—Origin of life. Astrobiology 17, 834–839.

## 1. Introduction

To introduce our argument and guide the discussion, it is essential to make explicit a set of assumptions. We will begin by noting that microorganisms on Earth can survive in the deep, cold ocean in the absence of light energy. For these life-forms, the reducing power of hydrogen sulfide or dissolved hydrogen gas is used as a source of electrons. An electron transport chain allows the electrons to fall through the redox potential to an acceptor, thereby releasing chemical energy that drives metabolism. The acceptor is usually molecular oxygen, a product of photosynthesis, but can also be any of a variety of other acceptors such as ferric iron, nitrate, sulfate, and in the case of methanogens, carbon dioxide. Given an energy source, together with sources of carbon and nitrogen, it seems possible that microorganisms could survive in the oceans of Enceladus and Europa.

But could microbial life originate on an ice-covered ocean world? To gauge the probability, we will describe two conditions that have been proposed as plausible sites for the origin of life on Earth, then consider whether life could emerge if similar conditions exist on Enceladus and Europa.

Our argument has a foundation of five observations and assumptions about the physical nature of Enceladus, as follows:

• There is a deep subsurface ocean on Enceladus, at least 10 km thick under the south polar terrain and covered by a layer of ice tens of kilometers thick but perhaps thinner at the south pole.
• Enceladus has abundant tidal heat (Lainey *et al.,* 2012) that is sufficient to maintain a long-lived liquid ocean.
• Sustained eruptions in the ocean floor break through the south pole ice and produce the plumes of frozen vapor mixtures (water, CO_2_, CH_4_, NH_3_, and other compounds) that were detected by Cassini.
• The plumes contain compounds consistent with the presence of hydrothermal sites on the ocean floor that are powered by chemical reactions. If so, the effluents are likely to be similar in properties (pH, temperature, composition, and origin) to the Lost City hydrothermal vents, with energy made available by serpentinization (Kelley *et al.,* 2001).
• The ocean is salty and may contain organic compounds that resemble the mixture of organic compounds in carbonaceous meteorites.

To further constrain our argument, we will assume seven conditions that must be met for life to begin:

(1) A mechanism exists by which dilute solutions of organic compounds can be sufficiently concentrated to undergo chemical reactions, and particularly condensation leading to polymer synthesis.
(2) The condensation reactions must be thermodynamically feasible.
(3) Polymers resembling peptides and nucleic acids can be synthesized by condensation reactions. The polymers must have the potential to act as catalysts and carriers of genetic information.
(4) Amphiphilic compounds can spontaneously assemble into closed, vesicular compartments.
(5) The polymers can be encapsulated in membrane-bounded vesicles to form protocells.
(6) There must be a source of nutrients and chemical energy available to support metabolism.
(7) Cycling environments, as opposed to equilibrium or steady state conditions, impose selective pressures on populations of protocells.

Hydrothermal sites are the only environments that can provide this suite of conditions. Other proposed environments such as a global ice sheet (Bada *et al.,* 1994), clay minerals (Ferris, 2002), and iron-sulfur minerals (Wächtershäuser, 1992) lack one or more of the above properties.

## 2. Hydrothermal Conditions in Terrestrial Geology

Soon after submarine hydrothermal vents (“black smokers”) were discovered, it was proposed that not only do they harbor an ecosystem based on microbial populations using hydrogen sulfide and hydrogen as energy sources, but they may also have been sites conducive for the origin of life (Corliss *et al.,* 1981; Baross and Hoffman, 1985; Russell *et al.,* 1993; Russell and Hall, 1997; Zierenberg *et al.,* 2000; Kelley *et al.,* 2001). Black smokers are associated with volcanism in which heat from an underlying magma plume brings circulating seawater to temperatures exceeding 400°C. When the heated water emerges from the vent, the dissolved minerals precipitate as a black cloud of iron sulfide and form chimneys.

A second version of hydrothermal vents is driven by serpentinization rather than volcanic heat (Kelley *et al.* 2005). When olivine is exposed to seawater, mineral components in the olivine (forsterite and fayalite) undergo an exothermic reaction that produces hydrogen gas and heat. The water becomes very alkaline (pH 10–11), and when it emerges from the vent the dissolved carbonate and hydroxide content precipitates as long-lived mineral chimneys.

Because the Cassini mission detected plumes of ice particles erupting hundreds of kilometers into space through the ice cover of Enceladus, it has been proposed that Enceladus, and perhaps Europa, may have environments resembling oceanic hydrothermal vents (Hsu *et al.,* 2015). Furthermore, given the assumption that life can begin in hydrothermal vent conditions, a reasonable speculation is that life could also originate on these ocean worlds and may still exist. For this reason, missions are being planned to send spacecraft through the Enceladus plumes to collect microgram amounts of plume components and then use ultrasensitive biosensors to analyze the sample for biosignatures.

How plausible is it that life can begin in hydrothermal conditions? A good way to sharpen the argument is to compare hydrothermal vents with an alternative site, the “warm little pond” first envisaged by Charles Darwin (1871). Unlike the salty seawater bathing hydrothermal vents and probably composing the oceans of Enceladus and Europa, the alternative site is characterized by precipitation on volcanic landmasses that produces freshwater hydrothermal fields with geysers, hot springs, and clay-lined freshwater pools. Furthermore, in contrast to the single water-mineral interface of hydrothermal vents, hydrothermal fields have three interfaces: atmosphere/water, atmosphere/mineral, and mineral/water. The freshwater pools undergo wetting and drying cycles that may be essential for the emergence of life on a habitable planet. In the rest of this discussion, we will compare the two hydrothermal systems in terms of their ability to promote processes that were likely to be initial stages on the pathway to cellular life.

## 3. Concentration Mechanisms

To undergo chemical reactions, dilute organic compounds must be sufficiently concentrated. One mechanism proposed for the hydrothermal vent environment is that thermal gradients in porous media can concentrate dilute solutes (Baaske *et al.,* 2007). This idea was tested by Herschy *et al.* (2014), who simulated the mineral structure of a vent with a porous alumina ceramic. A warm solution of dilute fluorescein dye was pumped into the cooled ceramic so that a 50°C thermal gradient was produced. When UV light was used to activate fluorescence, it was clear that the fluorescein had become concentrated in response to the thermal gradient, later estimated to be as much as 5000-fold.

Another laboratory simulation of hydrothermal concentration incorporated a dilute solution of fatty acids pumped through capillary channels in the presence of thermal gradient (Budin *et al.,* 2009; Budin and Szostak, 2010). The fatty acids were concentrated over 1000-fold, allowing assembly of membrane vesicles that encapsulated oligonucleotides in the mixture.

Although these results are consistent with thermal gradient concentration of dilute solutes, it remains to be seen whether this could actually occur in seawater circulating through vent minerals. In contrast, hydrothermal volcanic fields have an obvious concentrating mechanism. Small pools form and evaporate due to precipitation, and water levels fluctuate in response to geyser and hot spring activity. As water evaporates, films of solutes deposited on mineral surfaces can undergo extreme concentration. Because water activity has been markedly reduced, chemical potential is generated that can drive polymerization of monomers such as amino acids and nucleotides.

## 4. Thermodynamic Feasibility of Condensation Reactions

Barge *et al.* (2017) explored thermodynamic aspects of chemical reactions that could occur in hydrothermal vents. “We argue that life only emerges when and where particular planetary-scale conditions of chemical disequilibria are produced through the interactions of the atmosphere-hydrosphere complex with fresh mafic to ultramafic oceanic crust continually replenished by active partial melting of the mantle.” Their argument focuses on the energy made available by chemical reactions that produce reducing power such as dissolved hydrogen, which in turn drives reactions related to putative metabolic pathways that could be incorporated into early life. It was also proposed that pH gradients across mineral membranes may serve as an energy source for chemiosmotic synthesis of pyrophosphate bonds. Their discussion is largely confined to the chemistry of mineral-water interfaces and does not incorporate condensation reactions required for polymerization.

Because the essential polymers of life are nucleic acids and proteins, we assume that, in order for life to begin, there must have been a condensation mechanism by which ester and peptide bonds could have been synthesized in the absence of enzymatic catalysis. Condensation is an endergonic reaction, meaning that it is thermodynamically unfavorable in an aqueous solution and the spontaneous hydrolysis reaction is favored instead. Hydrothermal vents occur in seawater, so condensation and polymerization reactions face a significant uphill thermodynamic barrier that requires chemical activation to overcome. Plausible activation mechanisms have not yet been demonstrated that would support polymerization in hydrothermal vent conditions.

In contrast, the hydration-dehydration cycles that are ubiquitous in hydrothermal fields represent a significant source of physical energy. Because the energy driving evaporation is heat, evaporation is endergonic, and potential reactants become increasingly concentrated as evaporation takes place. During this process, water activity is reduced to the point that ester or peptide bonds can be synthesized by condensation reactions that link monomers into polymers in the crowded molecular film. In other words, the physical energy of evaporation and dehydration is transduced into chemical potential capable of driving condensation reactions.

The kinetics and thermodynamics of polymerization under these conditions have been investigated by numerical analysis by using Kintecus software (Ross and Deamer, 2016) in which variable values for the Gibbs free energy were inserted together with a known rate constant for hydrolysis. The free energy values chosen were +3.5, −3.5, and −10 kcal/mol, and the rate constant was 10^−6^ s^−1^, corresponding to the reported value for the dinucleotide uridyl-uridylic acid at pH 2 and *T* = 90°C. The results demonstrate that if mononucleotides are concentrated 1000-fold by drying and Δ*G* is negative, extensive polymerization could occur. Significantly, the synthesis rate is orders of magnitude faster than hydrolysis, which means that polymers will accumulate in the reaction mixture in a kinetic trap.

## 5. Polymer Synthesis

Kimura and Kitadai (2015) performed a theoretical analysis of the free energy available to drive peptide condensation reactions at the very low surface temperatures of Europa. They concluded that at temperatures below 118 K, the Gibbs energy becomes negative, which means, for instance, that diglycine can be synthesized from glycine on Europa and other icy moons. Even though a condensation reaction may be theoretically spontaneous, other factors will determine whether significant yields are possible. For instance, any chemical reaction requires activation energy to proceed, which in turn governs kinetics, so yields of peptide bonds might be vanishingly small at 118 K. Another consideration is that reactants must be able to diffuse in order to react, and diffusion in ice will be extremely slow at such temperatures.

Temperature and activation energy are less of a concern in hydrothermal environments, but the Gibbs energy must still be negative, which is not easy to achieve in aqueous conditions. Therefore, yields are expected to be small even when reactants are reasonably concentrated. For instance, Burcar *et al.* (2015) detected small yields of dimers when 15 m*M* AMP was circulated in a laboratory simulation of a hydrothermal vent, but longer oligomers up to four nucleotides were only synthesized if a chemically activated phosphoimidazole ester of AMP was added.

Much longer polymers (10 to >100 nucleotides in length) are produced in laboratory simulations in which 5'-mononucleotides were exposed to cycles of hydration and dehydration at acidic pH ranges and elevated temperatures characteristic of fluctuating pools in hydrothermal fields. Rajamani *et al.* (2008) employed multilamellar lipid membranes as organizing agents to promote polymerization and reported that polymers ranging up to 100 nucleotides in length were synthesized. The products could be labeled by the enzyme T4 kinase, which transferred a labeled phosphate group from ATP to the ends of the RNA-like polymers. The labeled products could then be visualized as bands by gel electrophoresis. DeGuzman *et al.* (2014) and Da Silva *et al.* (2015) found that the polymers could also be stained by ethidium bromide, a dye that binds to nucleic acids, and products ranging from 10 to >100 nucleotides were again observed as fluorescent bands in nondenaturing agarose gels.

The polymers were also detected by nanopore analysis (DeGuzman *et al.,* 2014), which confirmed that polyanionic chains resembling single-stranded RNA were present in the mixture. These results support the conclusion that polymerization can be driven by conditions simulating hydration-dehydration cycles occurring in hydrothermal fields.

## 6. Assembly of Amphiphilic Compounds into Membranous Vesicles

An essential requirement for the origin of cellular life is the availability of amphiphilic compounds and conditions that permit such compounds to assemble into the boundary membranes of primitive cells. *Amphiphile* is a general term referring to molecules composed of a nonpolar hydrophobic moiety, usually a hydrocarbon chain, with a polar hydrophilic group at one end of the molecule. Because the hydrophobic portion of an amphiphile is insoluble in water and the hydrophilic group interacts strongly with water, certain amphiphiles are able to assemble into bilayer structures that form the boundaries of all cellular life today. The simplest amphiphiles are carboxylic acids, basically a hydrocarbon chain with a carboxyl group at one end. Carboxylic acids up to 13 carbons in length are present in carbonaceous meteorites and have been demonstrated to assemble into membranous vesicles. (See Deamer *et al.,* 2002, for a review). For this reason, they are often used in simulations of prebiotic protocellular compartments (Budin *et al.,* 2014).

**FIG. 1.**
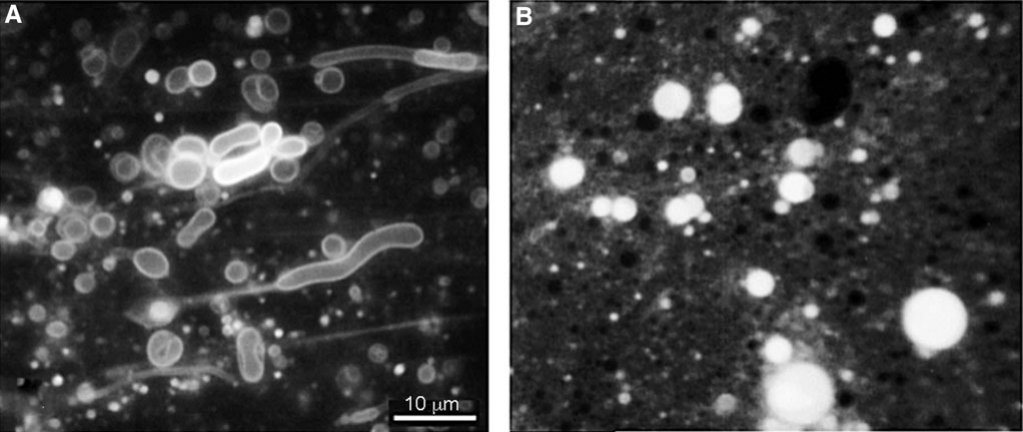
(**A**) Membranous vesicles spontaneously form when a mixture of fatty acid and fatty alcohol is dispersed in water (Monnard and Deamer, 2002). If the same mixture is exposed to a single evaporation-rehydration cycle in the presence of short strands of duplex DNA stained with acridine orange, a fluorescent dye, the concentrated polymer is encapsulated within membrane-bounded vesicles (**B**).

Phospholipids are the primary components of most cell membranes and contain two carboxylic acids attached by ester bonds to glycerol phosphate, which in turn is esterified to head groups like choline, ethanolamine, serine, and glycerol. When dispersed in dilute solutions of ionic solutes, carboxylic acids and phospholipids spontaneously self-assemble into microscopic membranous vesicles bounded by bilayer structures (Fig. 1). However, high concentrations of divalent cations in seawater tend to inhibit membrane assembly from pure amphiphilic compounds like monocarboxylic acids (Monnard and Deamer, 2002). Furthermore, the sodium chloride concentration in seawater is ∼0.6 *M,* and this can exert osmotic pressure that inhibits the ability of membranous vesicles to form. Because of these ionic effects on membranes, life today maintains intracellular calcium ion concentrations at sub-micromolar levels and actively transports sodium ions outward in exchange for potassium ions to maintain an intracellular KCl concentration of ∼100 m*M*. Mulkidjanian *et al.* (2012) argued that this is consistent with an origin of life in hydrothermal fields rather than hydrothermal vents in seawater.

Self-assembly of membranes composed of amphiphiles has not been demonstrated in hydrothermal vents, either *in situ* or in laboratory simulations. In contrast, hydrothermal fields are supplied by what is essentially distilled water with ionic solutes in millimolar ranges that are more conducive to the self-assembly of amphiphiles into vesicles.

## 7. Encapsulation of Polymers

Because organic compounds in solution become highly concentrated films on dry mineral surfaces, amphiphilic compounds fuse upon drying to form a multilamellar film that effectively sandwiches and concentrates solutes between lipid layers (Toppozini *et al.,* 2013). A subsequent rehydration cycle produces large lipid vesicles that have encapsulated a significant fraction of the solutes (Shew and Deamer, 1985). Dehydration-rehydration cycles are the simplest process by which polymers can be encapsulated in membranous vesicles (Fig. 1B) and would obviously be a common occurrence in hydrothermal fields. Because hydrothermal vents cannot undergo such cycles, how protocells with encapsulated polymers could assemble is an open question.

## 8. Sources of Nutrients and Chemical Energy

Gaidos *et al.* (1999) and Jakosky and Shock (1998) analyzed possible sources of energy on Europa that might be available to maintain microbial life. Both papers concluded that, because light energy could not penetrate the kilometer-thick ice covering a global ocean, the only available energy would be that present in certain mineral combinations. On Enceladus, the presence of Si-containing nanoparticles in the plume, and the vent conditions inferred from it, suggest serpentinization as a possible source of chemical energy. In the absence of chemical energy, life could not be sustained even if molecular systems capable of growth, replication, and metabolism assembled from organic compounds present in the ocean.

Russell *et al.* (2014) explored the potential for linked chemical reactions to emerge in hydrothermal vents, pointing out that the relative abundance of dissolved hydrogen gas in vent fluids could supply a source of reducing power. They envisaged an elaborate set of putative metabolic pathways that could occur within a mineral compartment analogous to a cellular compartment. Herschy *et al.* (2014) investigated the possibility that alkaline hydrothermal vents might support the reduction of carbon dioxide to biologically significant reduced species. Their approach was to expose dissolved carbon dioxide at an acidic pH range to reducing power in the form of ferrous iron. To simulate an alkaline vent, a solution of K_2_HPO_4_, Na_2_Si_3_O_7_, and Na_2_S at pH 11 was slowly pumped through an orifice into an acidic solution of FeCl_2_, NaHCO_3_, and NiCl_2_. The precipitates of ferrous silicates and phosphates that formed resembled the mineral structures associated with vents. After several hours, small amounts of formic acid were detected (50 μ*M*), and in some of the runs traces of formaldehyde (∼100 n*M*).

There have been no similar studies related to simulations of primitive metabolism in hydrothermal fields. However, prebiotic hydrothermal fields could be supplied with nutrient solutes from a continuing infall of interplanetary dust particles (Chyba and Sagan, 1992) or from geochemical synthesis associated with volcanism (Cody *et al.,* 2001). The nutrients would accumulate and become concentrated in freshwater ponds on volcanic land masses.

## 9. Competition, Selection, and Evolution

There is no doubt that hydrothermal vents provide a source of disequilibrium and chemical energy, but it is unclear how encapsulated polymers could emerge under these conditions, nor is it obvious what selective factors would come into play. We have argued that hydration-dehydration cycles commonly occurring in hydrothermal fields not only provide energy for condensation reactions but also present sequential environmental stresses that initiate selection and evolution within protocell populations (Damer and Deamer, 2015; Damer, 2016).

## 10. Summary

The submarine hydrothermal vents and volcanic hydrothermal fields that have been proposed as alternative sites for the origin of life on Earth can guide the development of future flight missions designed to sample icy plumes of Enceladus, and future landers on Enceladus or Europa. It is essential to have more detailed information about the properties of hydrothermal conditions and their potential to promote physical and chemical processes related to the origin of life. To that end, we propose the following questions to be addressed *in situ,* or by using laboratory simulations of the alternative sites described here:

• Can dilute solutes become sufficiently concentrated to undergo chemical reactions?
• Can amphiphiles assemble into membranous compartments over the range of temperatures, salt concentrations, and pH values related to each site?
• Will chemical energy available in the environment drive reactions such as carbon fixation or polymerization?
• Will products of reactions accumulate within the site rather than dispersing into the bulk phase environment?
• Are biologically relevant polymers synthesized with chain lengths sufficient to act as catalysts or incorporate genetic information?
• Is there a plausible physical mechanism that can produce encapsulated polymers as protocells and subject them to combinatorial selection?

Even though there is no consensus yet about which of the two sites has more explanatory power, it is possible to use what we know so far as a way to judge the probability that life can begin in an ice-covered liquid ocean. Given the assumption that life can originate in hydrothermal vent conditions on Earth, and that similar conditions exist on Enceladus, it is feasible that life could be present in its ocean as well as that of Europa. Furthermore, depending on the abundance of microbial life in the ocean, it might be possible to detect biosignatures in plume samples.

However, based on the weight of evidence described here, it seems equally plausible that the origin of life may require cycles of hydration and dehydration in fluctuating environments such as hydrothermal fields. Due to the lack of volcanism and associated land masses that are common on Earth, future missions may find that Enceladus and Europa are habitable but lifeless. Of course, one might imagine that life began on Earth and then over three billion intervening years was delivered to Enceladus on meteorites produced by giant impacts on Earth. In the argument presented here, for lack of evidence, we have discounted distribution of life by a limited version of panspermia.

There is strong evidence that Mars once had a shallow ocean and hydrothermal conditions related to volcanism, so a reasonable speculation is that microbial life could originate in hydrothermal fields on Mars and may still survive in deep subsurface brines, just as halophilic extremophiles thrive in such conditions on Earth. It seems likely that in the next two decades future probes and landers will provide evidence that reduces speculations to certainties: Life will either be confined to Earth, or we will find it elsewhere in the Solar System.
